# Peritumoral Infiltration of Regulatory T Cells Reduces the Therapeutic Efficacy of Bacillus Calmette–Guérin Therapy for Bladder Carcinoma In Situ

**DOI:** 10.1111/iju.70044

**Published:** 2025-03-14

**Authors:** Yusuke Fukiage, Akifumi Muramoto, Naoki Terada, Motohiro Kobayashi

**Affiliations:** ^1^ Department of Tumor Pathology, Faculty of Medical Sciences University of Fukui Eiheiji Japan; ^2^ Department of Urology, Faculty of Medical Sciences University of Fukui Eiheiji Japan

**Keywords:** BCG vaccine, carcinoma in situ, regulatory T cell, tumor microenvironment, urinary bladder

## Abstract

**Objectives:**

Intravesical instillation of bacillus Calmette–Guérin (BCG) is the standard treatment for bladder carcinoma in situ (CIS); however, factors that predict its therapeutic efficacy have not been identified. We focused on immune cells infiltrating within 20 μm of tumor cells and examined factors that predict the efficacy of intravesical BCG treatment.

**Methods:**

Formalin‐fixed, paraffin‐embedded tissue specimens from 82 patients with bladder CIS treated with intravesical BCG were used. Patients who relapsed after BCG treatment were grouped as non‐responders, and those who did not were grouped as responders. Tissue sections were immunostained for CD4, CD8, and forkhead box P3 (FOXP3), a marker of regulatory T cells (Tregs). The number of immune cells positive for the above markers present within 20 μm of the lower edge of the basement membrane on which CIS is present was counted and compared between groups.

**Results:**

Both the peritumoral Treg density and Treg+/CD4+ cell ratio were significantly greater in nonresponders than in responders. The patients were divided into high and low groups based on Treg density and Treg+/CD4+ cell ratio cut‐off values; recurrence‐free survival was significantly longer in the low group than in the high group (*p* = 0.005 and *p* < 0.001, respectively).

**Conclusions:**

The Treg density and Treg+/CD4+ cell ratio within 20 μm of bladder CIS may be useful predictors of therapeutic response to BCG.

**Abbreviations**
AUC: area under the curve
BCG: Bacillus Calmette–Guérin
CI: confidence interval
CIS: carcinoma in situ
FOXP3: forkhead box protein 3
HR: hazard ratio
MIBC: muscle invasive bladder cancer
NMIBC: non‐muscle invasive bladder cancer
PD‐1: programmed cell death 1
RFS: recurrence‐free survival
TURBT: transurethral resection of bladder tumor

## 1 Introduction

Bladder cancer is the 12th most common cancer in the world, with 75% of cases being non‐muscle invasive bladder cancer (NMIBC) at initial diagnosis [1, 2]. Bladder carcinoma in situ (CIS), accounting for 19% of NMIBC cases, is a high‐grade cancer confined to the mucosa; if left untreated, it often progresses to muscle invasive bladder cancer (MIBC) [3, 4, 5]. Additionally, bladder CIS is often multicentric, making complete transurethral resection difficult. Therefore, intravesical administration of bacillus Calmette–Guérin (BCG) is the gold standard treatment for bladder CIS. Although intravesical BCG administration has a high response rate for bladder CIS, some cases show recurrence or progression to MIBC, and predictive factors remain poorly understood [5].

Regulatory T cells (Tregs), whose marker is forkhead box protein 3 (FOXP3), are a subset of CD4‐positive T cells that suppressively regulate immune responses and maintain immune homeostasis. In the tumor microenvironment, Tregs generally promote tumor growth [6]. A high number of FOXP3‐positive T cells around bladder tumors was associated with a poor response to BCG treatment for NMIBC [7]. However, contrasting reports exist; some reports have suggested that the presence of FOXP3‐positive T cells around bladder tumors is associated with a favorable patient prognosis [8], whereas others found no significant relationship between the presence of these cells and patient outcomes [9]. Thus, the impact that FOXP3‐positive lymphocytes present around bladder cancer have on patient prognosis remains unclear.

Recently, the distance between immune and cancer cells has been noted as predictors of patient prognosis [10, 11]. Anti‐programmed cell death 1 (PD‐1) antibody therapy for melanoma is more effective when CD8‐positive T cells infiltrate within 20 μm of the tumor cells [12]. However, studies on the effects of BCG treatment for bladder cancer have not focused on the spatial relationship between the tumor and peritumoral immune cells, and the definition of “peritumoral” is often ambiguous [7, 13]. Moreover, few studies have examined the relationship between immune cells and the efficacy of BCG treatment focused solely on bladder CIS.

Here, we aimed to evaluate the relationship between immune cells in the peritumoral region and the efficacy of BCG treatment by performing quantitative immunohistochemical analyses of bladder CIS. We clearly defined the peritumoral region and determined the numbers of various immune cells within that region prior to BCG treatment, as well as examined the correlation between these cell counts and the clinical outcome of the patient. Our findings indicate that FOXP3‐positive cell density and FOXP3+/CD4+ cell ratio may serve as biomarkers to predict the therapeutic efficacy of BCG in bladder CIS.

## 2 Methods

### 2.1 Patients

This study included patients who: (i) underwent transurethral resection of bladder tumor (TURBT) at the University of Fukui Hospital (Fukui, Japan) and one affiliated hospital between April 2006 and March 2020, (ii) were pathologically diagnosed with bladder CIS or NMIBC with CIS, and (iii) received first BCG treatment postoperatively. Intravesical BCG treatment consisted of weekly instillations of Immunobladder (BCG Tokyo 172 strain; Japan BCG Research Institute, Tokyo, Japan) or ImmuCyst (Connaught strain; Sanofi, Paris, France) for 6–8 consecutive weeks as induction therapy. BCG maintenance therapy was generally scheduled at 3, 6, 12, 18, and 24 months after the initial BCG instillation, with weekly instillations for 3 consecutive weeks. Patients (i) with less than six BCG instillations, (ii) diagnosed with upper urinary tract tumors before or after BCG treatment, or (iii) with a follow‐up period of less than 2 years were excluded. Consequently, 82 patients were enrolled in this study. Intravesical chemotherapy, defined in this study as six or more instillations of pirarubicin (THP), was not administered after CIS diagnosis. However, 13 secondary onset patients had intravesical chemotherapy before CIS diagnosis. Patients were classified as non‐responders or responders according to the presence or absence of recurrence after BCG treatment, respectively. Patients were followed up with cystoscopy and urine cytology every 3 months within the first 2 years of treatment, and at least every 6 months thereafter, with computed tomography scans every 1–2 years for upper urinary tract evaluations. Recurrence was defined as pathologically confirmed intravesical recurrence in reoperation specimens or the presence of metastases confirmed by imaging studies. The date of recurrence was defined as the date of reoperation or the date confirmed by imaging. New onset refers to the initial diagnosis of bladder CIS and secondary onset to the diagnosis of bladder CIS in a patient previously identified as having NMIBC excluding CIS.

### 2.2 Immunohistochemistry

Formalin‐fixed, paraffin‐embedded tissue blocks of bladder tumors removed via transurethral surgery prior to BCG treatment were obtained from the pathology archives of the hospital and used for immunohistochemistry. The following monoclonal antibodies served as primary antibodies: EPR6855 (rabbit IgG; Abcam, Cambridge, UK) recognizing CD4, C8/144B (mouse IgG; Abcam) recognizing CD8, and 236A/E7 (mouse IgG; Abcam) recognizing FOXP3. Immunostaining with these antibodies was conducted using the Histofine system (Nichirei Biosciences, Tokyo, Japan), as described previously [14, 15]. Additionally, double immunostaining for CD4 and FOXP3 was conducted in 10 cases, with appropriate modifications to previously described methods [16].

### 2.3 Quantification of Immunohistochemistry

**FIGURE 1:**
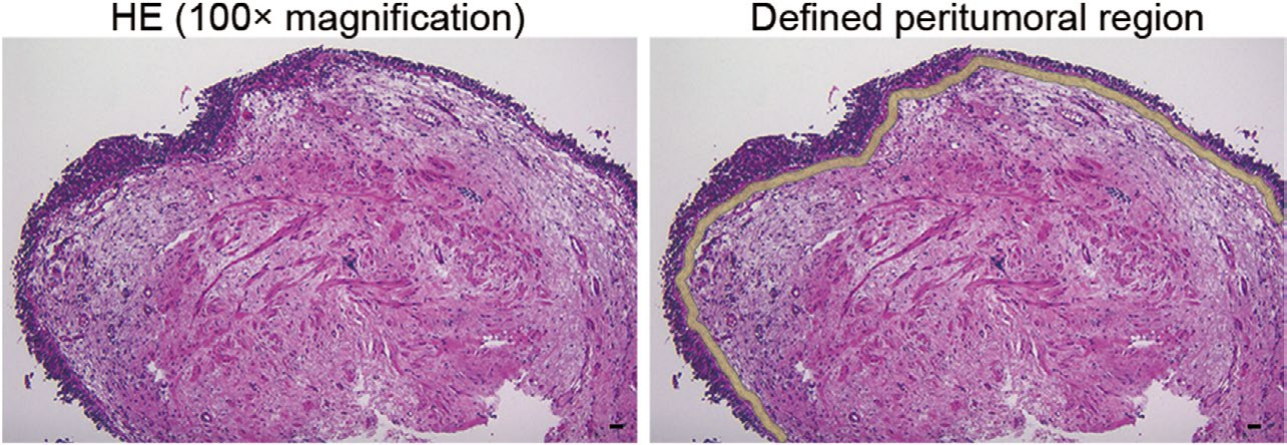
Representative histology of bladder carcinoma in situ (CIS) and the defined peritumoral region used for analysis. The left panel shows hematoxylin and eosin (HE) staining at x100 magnification. The right panel displays the defined peritumoral region, in which a 20 μm area from the lower edge of the basement membrane is highlighted in yellow. This region was used for quantification of CD4^−^, CD8^−^, and FOXP3‐positive cells. Scale bar = 20 μm.

Immunostained tissue sections were observed under an AX‐80 microscope (Olympus, Tokyo, Japan) and photographed at x100 magnification using an FX380 digital camera (Olympus). Images were analyzed using the image filing software, FLVFS‐LS ver.1.12 (Olympus). We first measured the area from the lower edge of the basement membrane, on which bladder CIS was present, to a depth of 20 μm in the captured image (Figure 1). Since the extent of CIS varied among specimens, the measurement width was determined based on the actual area where CIS was present, rather than being fixed. We then determined the number of CD4‐, CD8‐, and FOXP3‐positive cells in the corresponding area of the serial sections. Cell density was calculated by dividing the number of cells positive for each marker by the area of the region. For cases with multiple CIS‐containing specimens, measurements were taken from each, and the average value was used. If only one specimen contained CIS, a single field was measured.

### 2.4 Statistical Analysis

Patient characteristics were analyzed using Student's *t*‐test for age; chi‐squared test for T stage, diagnosed condition, smoking history, and prior intravesical chemotherapy; and Fisher's exact test for sex and BCG maintenance therapy. Differences in the number of CD4‐, CD8‐, and FOXP3‐positive cells, and in the FOXP3+/CD4+ cell ratio between groups were analyzed using Student's *t*‐test. Receiver operating characteristic (ROC) curve analysis was performed to determine the cut‐off values for FOXP3‐positive cell density and FOXP3+/CD4+ cell ratio. The area under the curve (AUC) was calculated, and cut‐off values were established based on the shortest distance to the upper‐left corner of the ROC curve. Recurrence‐free survival (RFS), calculated from the date of BCG treatment initiation, was analyzed using the Kaplan–Meier method and compared using the log‐rank test. Univariate and multivariate regression analyses of recurrence factors were performed using Cox's proportional hazards model. Multivariate analysis included age, sex, T stage, diagnosed condition, smoking history, BCG maintenance therapy, prior intravesical chemotherapy, FOXP3‐positive cell density, and FOXP3+/CD4+ cell ratio. All analyses were performed using IBM SPSS Statistics 28 software (IBM, Armonk, NY). *p* < 0.05 were considered statistically significant.

## 3 Results

### 3.1 Characteristics of Responders and Non‐responders to BCG Treatment

First, we assessed whether differences existed in patient characteristics between responders and nonresponders. As shown in Table 1, no significant differences were observed between responders and nonresponders in age, sex, T stage, diagnosed condition, smoking history, BCG maintenance therapy, and prior intravesical chemotherapy. Thus, differences in patient characteristics were unlikely to affect the results.

**TABLE 1:** Patient characteristics.

	All patients	Responder	Non‐responder	*p*
	(*n* = 82)	(*n* = 50)	(*n* = 32)	
Age (years)	72	71.5	75.8	0.061
Sex				
Male	73	42	31	0.067
Female	9	8	1	
T stage				
Tis	44	25	19	0.406
Ta/T1 with Tis	38	25	13	
Diagnosed condition				
New onset	47	30	17	0.539
Secondary onset	35	20	15	
Smoking				
Yes	54	30	24	0.162
No	28	20	8	
BCG maintenance therapy				
Yes	11	8	3	0.305
No	71	42	29	
Prior intravesical chemotherapy				
Yes	13	7	6	0.758
No	69	43	26	

### 3.2 Relationship Between Immune Cell Density and BCG Treatment Efficacy

**FIGURE 2:**
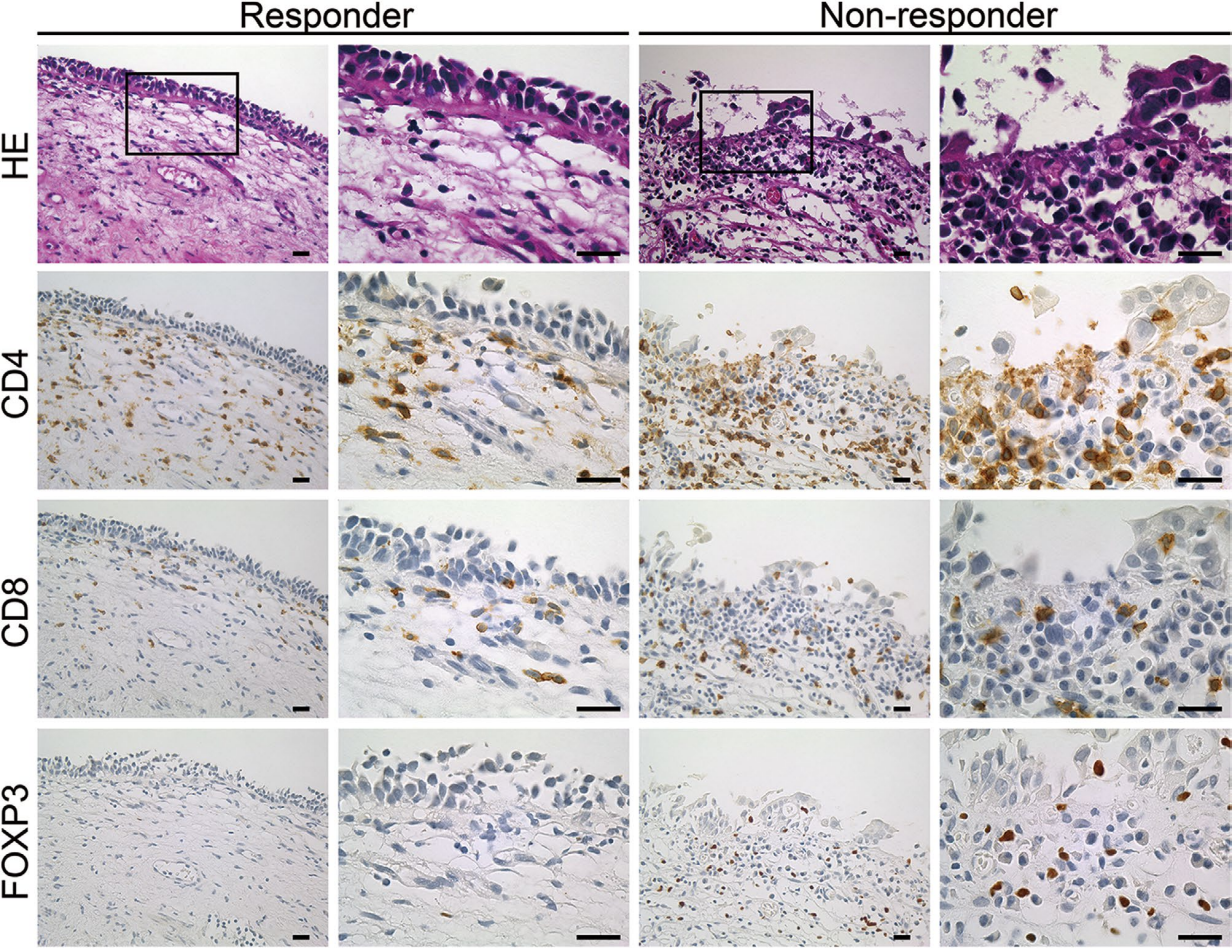
Representative histology of responders and non‐responders. Specimens were stained with hematoxylin and eosin (HE) or immunostained for CD4, CD8, and FOXP3. Signals were visualized with 3,3′‐diaminobenzidine (brown). The right column for each responder and non‐responder shows an enlarged view of the rectangle indicated in the left column. CD4‐positive cells are found in both responders and non‐responders; however, FOXP3‐positive cells are predominantly observed in non‐responders. Scale bar = 20 μm.

**FIGURE 3:**
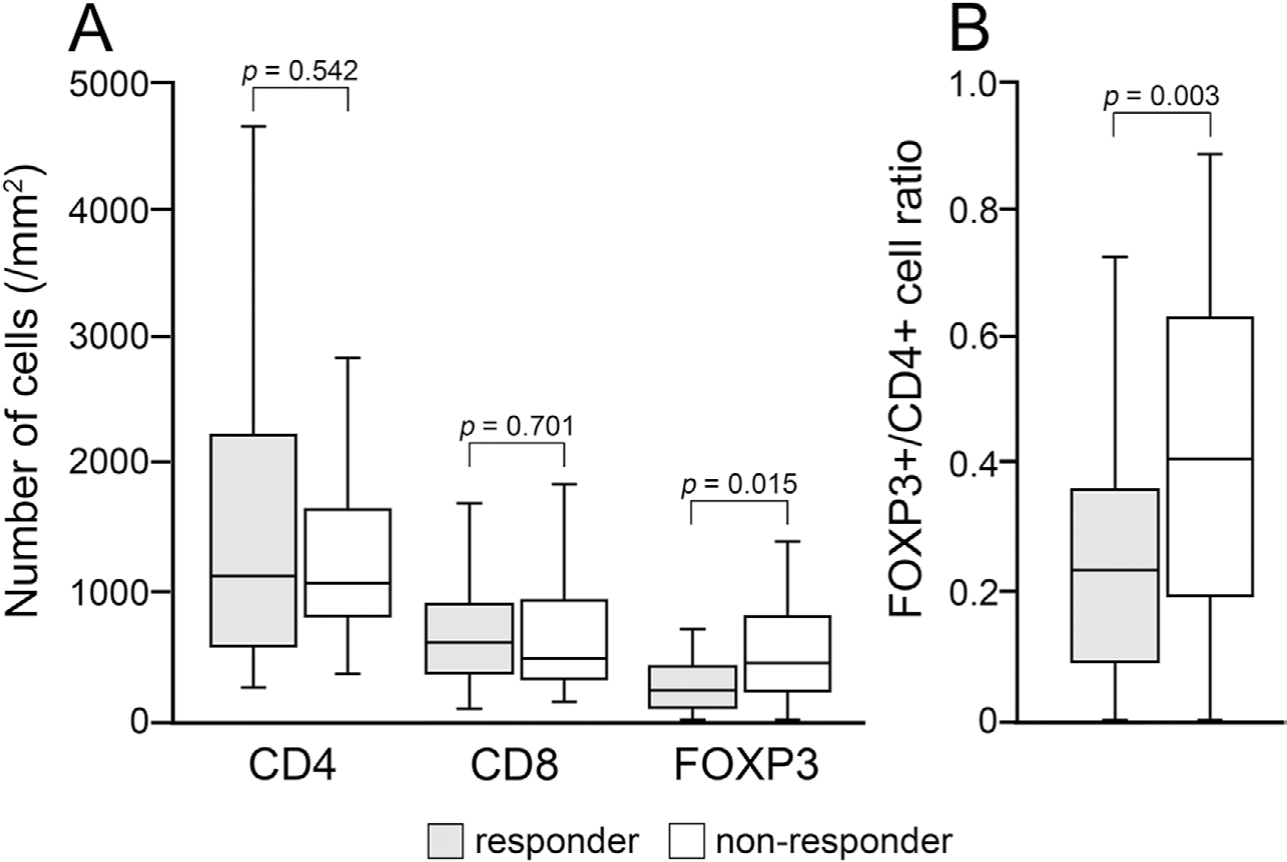
Comparison of the density of CD4‐, CD8‐, and FOXP3‐positive cells (A) and the FOXP3+/CD4+ cell ratio (B) between responders and non‐responders. The density of FOXP3‐positive cells and the FOXP3+/CD4+ cell ratio in non‐responders were significantly greater than those in responders. Box plots illustrate the medians and the 25th and 75th percentiles. In both the panels, gray squares indicate responders and white squares the non‐responders.

**FIGURE 4:**
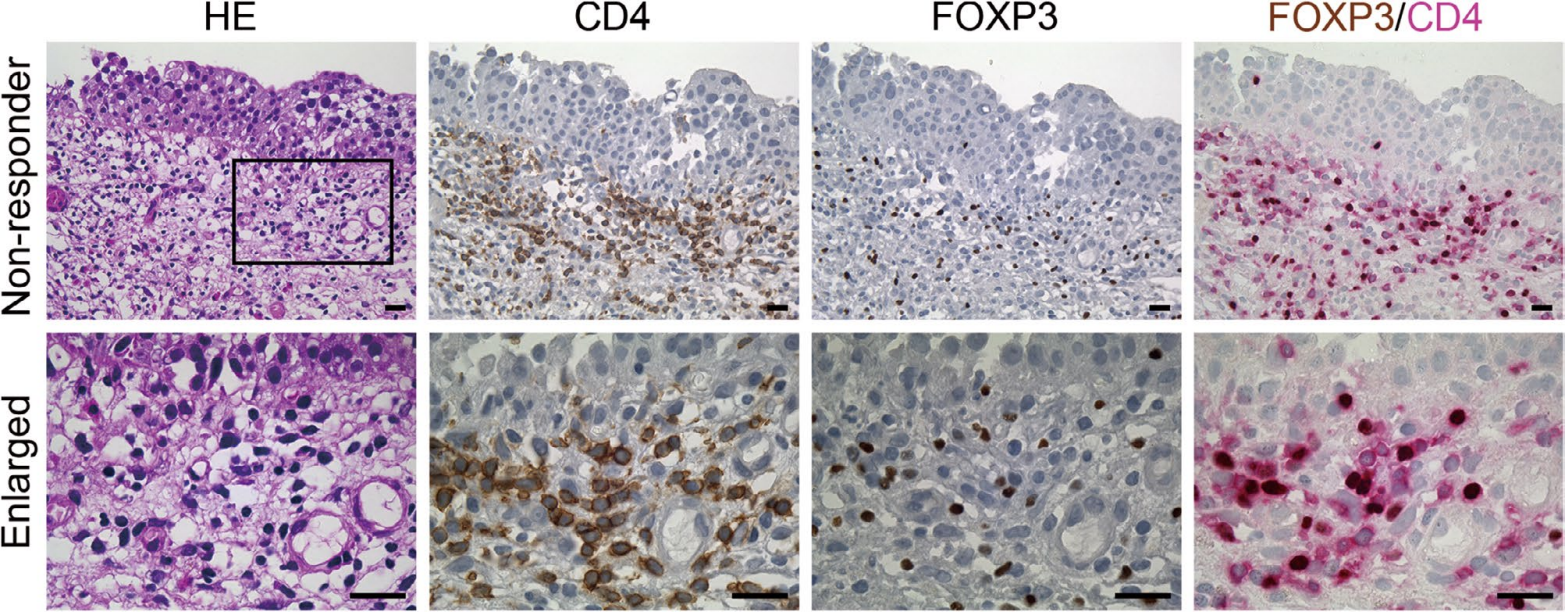
Immunohistochemical profiles of Treg cells infiltrated in the bladder mucosa of non‐responders. A representative case is shown. Signals were visualized with 3,3′‐diaminobenzidine (brown). In the case of double immunohistochemistry for FOXP3 and CD4, CD4 signals were visualized using Vulcan Fast Red Chromogen Kit 2 (Biocare Medical, Pacheco, CA). The bottom panels are enlarged images of the boxed region in the top left panel. Scale bars = 20 μm.

To evaluate the relationship between the number of CD4‐, CD8‐, and FOXP3‐positive cells infiltrating the mucosal lamina propria beneath the bladder CIS and BCG treatment efficacy, we quantified these immune cells (Figure 2). Prior intravesical chemotherapy had no significant effect on the densities of CD4‐, CD8‐, and FOXP3‐positive cells (Table S1). Consequently, the CD4‐positive cell density in responders (1506 ± 1181/mm^2^) and non‐responders (1356 ± 895/mm^2^) did not differ significantly (*p* = 0.542). The CD8‐positive cell density in responders (726 ± 530/mm^2^) and non‐responders (681 ± 501/mm^2^) did not differ significantly (*p* = 0.701) either. However, the FOXP3‐positive cell density was significantly greater in non‐responders (575 ± 536/mm^2^) than in responders (308 ± 326/mm^2^) (*p* = 0.015). Moreover, the FOXP3+/CD4+ cell ratio was greater in non‐responders (0.41 ± 0.27) than in responders (0.24 ± 0.18), with a high statistical significance (*p* = 0.003) (Figure 3). These results indicate that the peritumoral FOXP3‐positive cell density and FOXP3+/CD4+ cell ratio prior to BCG treatment are associated with treatment response. Additionally, double immunostaining for CD4 and FOXP3 in 10 representative cases confirmed that nearly all FOXP3‐positive cells were also positive for CD4 (Figure 4). Therefore, the density of CD4 + FOXP3+ double‐positive cells was likely to be close to that of FOXP3‐positive cells.

### 3.3 Establishment of Cut‐Off Values for the FOXP3‐Positive Cell Density and FOXP3+/CD4+ Cell Ratio

**FIGURE 5:**
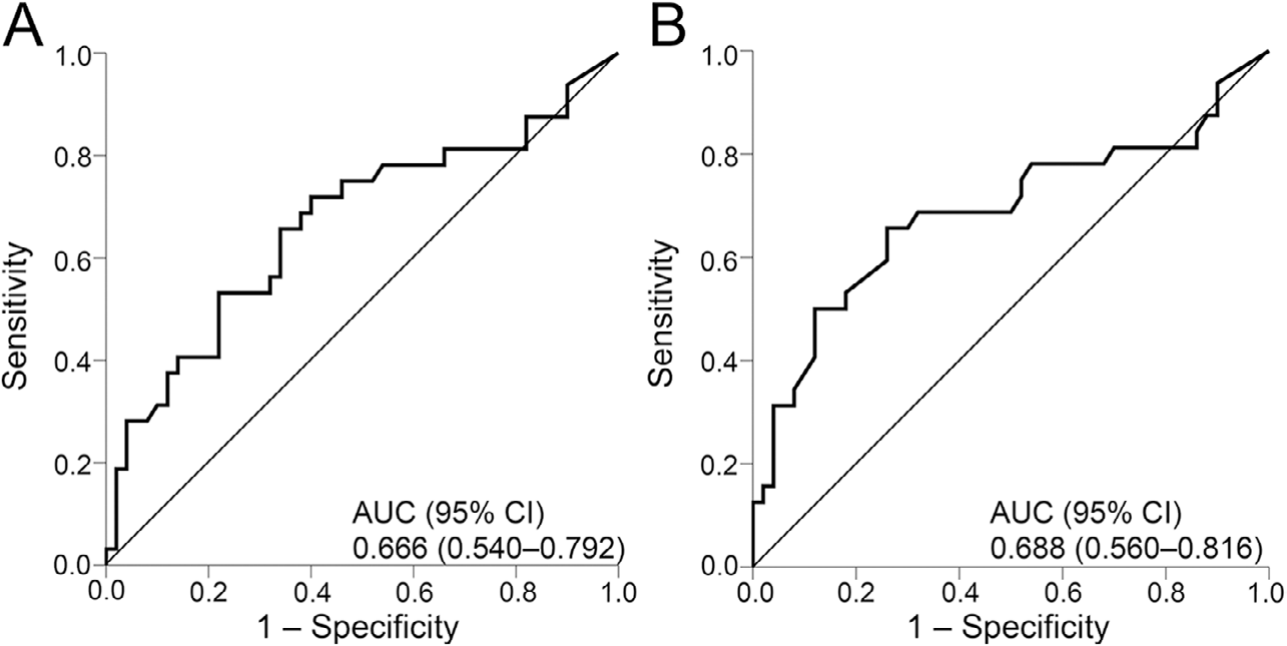
Receiver operating characteristic curves for the density of FOXP3‐positive cells (A) and the FOXP3+/CD4+ cell ratio (B) for the treatment effect of bacillus Calmette–Guérin. AUC, area under the curve; CI, confidence interval.

ROC curve analysis determined cut‐off values for FOXP3‐positive cell density (AUC = 0.66) and FOXP3+/CD4+ ratio (AUC = 0.68) (Figure 5). Cut‐off values for the FOXP3‐positive cell density and FOXP3+/CD4+ cell ratio were set at 300/mm^2^ and 0.33, respectively.

### 3.4 FOXP3‐Positive Cell Density May Be a Biomarker for Predicting BCG Treatment Efficacy

**FIGURE 6:**
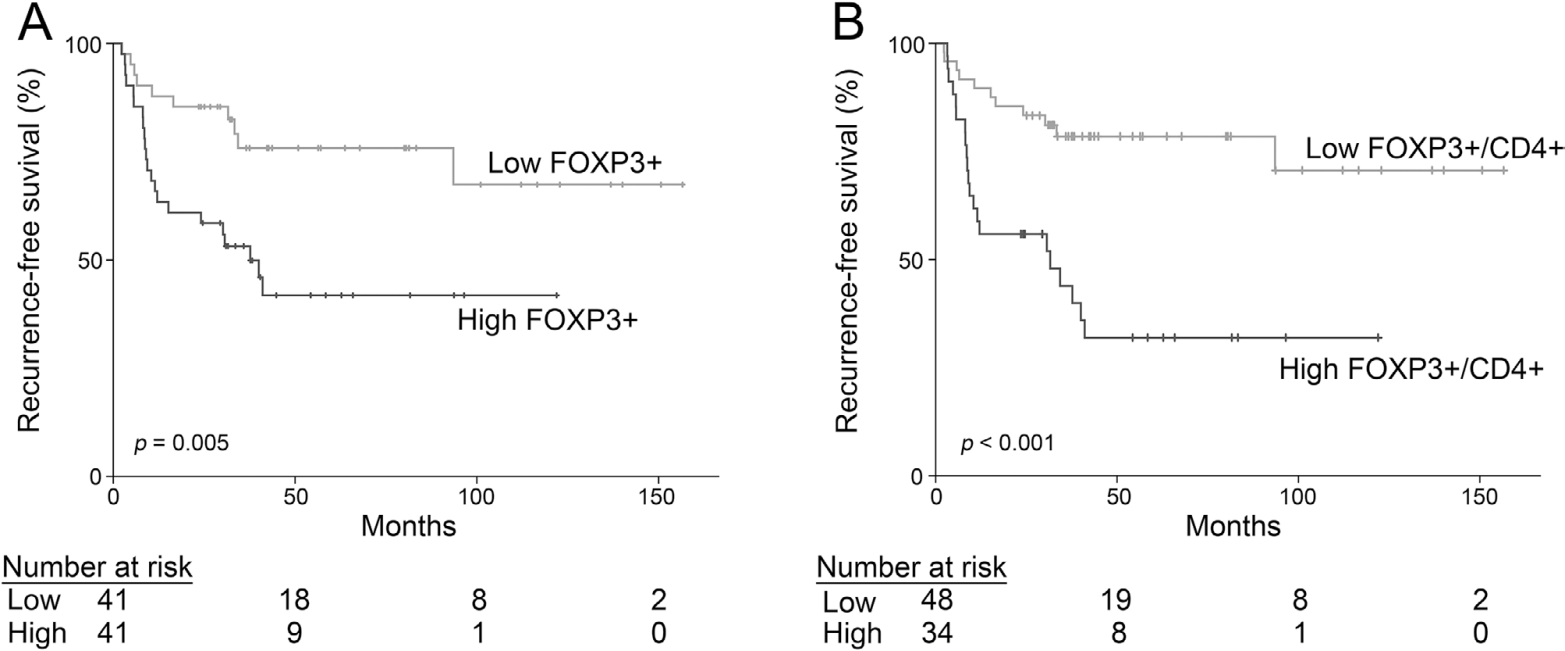
Kaplan–Meier curves for the density of FOXP3‐positive cells (A) and the FOXP3+/CD4+ cell ratio (B). In both analyses, the time to recurrence is significantly shorter in the high group than in the low group.

Using the above cut‐off value for the FOXP3‐positive cell density (300/mm^2^), we classified 82 patients into high (*n* = 41) and low (*n* = 41) FOXP3‐positive cell density groups and compared their characteristics. A significantly higher percentage of patients in the low FOXP3‐positive cell density group (22.0%) received BCG maintenance therapy compared with that in the high FOXP3‐positive cell density group (4.9%) (*p* = 0.024). No significant differences were observed for the other parameters (Table 2). Next, to examine the relationship between FOXP3‐positive cell density and time to relapse after BCG treatment, we conducted a Kaplan–Meier curve analysis with a log‐rank test. The mean RFS in the high FOXP3‐positive cell density group was 60.8 months, significantly shorter than that in the low FOXP3‐positive cell density group (117.7 months; *p* = 0.005) (Figure 6A). Recurrence patterns did not differ significantly between the two groups (*p* = 1.000) (Table S2). We then performed univariate and multivariate Cox regression analyses to explore factors associated with RFS after BCG treatment. Consequently, a high FOXP3‐positive cell density (≥ 300/mm^2^) was associated with a shorter RFS after BCG treatment in both univariate (hazard ratio [HR]: 2.81, 95% confidence interval [CI]: 1.33–5.96, *p* = 0.007) (Table 3) and multivariate analyses (HR: 3.62, 95% CI: 1.60–8.20, *p* = 0.002) (Table 4). These findings indicate that FOXP3‐positive cell density may be a potential biomarker for predicting BCG treatment efficacy.

**TABLE 2:** Patient characteristics (separated by cut‐off values).

	FOXP3‐positive cell density			FOXP3+/CD4+ cell ratio		
	Low	High	*p*	Low	High	*p*
	(*n* = 41)	(*n* = 41)		(*n* = 48)	(*n* = 34)	
Age (years)	73	73.3	0.901	73.1	73.2	0.97
Sex						
Male	38	35	0.241	42	31	0.441
Female	3	6		6	3	
T stage						
Tis	21	23	0.658	26	18	0.913
Ta/T1 with Tis	20	18		22	16	
Diagnosed condition						
New onset	21	26	0.264	28	19	0.825
Secondary onset	20	15		20	15	
Smoking						
Yes	29	25	0.352	31	23	0.773
No	12	16		17	11	
BCG maintenance therapy						
Yes	9	2	0.024^a^	7	4	0.49
No	32	39		41	30	
Prior intravesical chemotherapy						
Yes	7	6	1.000	5	6	0.133
No	34	35		43	24	

Abbreviation: FOXP3, forkhead box P3.

^a^ *p* < 0.05.

**TABLE 3:** Cox proportional hazard analysis for recurrence‐free survival after bacillus Calmette–Guérin treatment (univariate).

	Reference	HR (95% CI)	*p*
Age (years)			
≥ 72	< 72	1.70 (0.83–3.51)	0.145
Sex			
Male	Female	4.51 (0.62–33.04)	0.138
T stage			
Ta/T1 with Tis	Tis	0.75 (0.37–1.52)	0.428
Diagnosed condition			
New onset	Secondary onset	0.80 (0.40–1.60)	0.531
Smoking			
Yes	No	2.07 (0.92–4.62)	0.077
BCG maintenance therapy			
Yes	No	0.51 (0.16–1.69)	0.272
Prior intravesical chemotherapy			
Yes	No	1.533 (0.63–3.74)	0.348
FOXP3‐positive cell density			
≥ 300	< 300	2.81 (1.33–5.96)	0.007^a^
FOXP3+/CD4+ cell ratio			
≥ 0.33	< 0.33	3.78 (1.81–7.90)	< 0.001^a^

Abbreviations: CI, confidence interval; HR, hazard ratio.

^a^ *p* < 0.05.

**TABLE 4:** Cox proportional hazard analysis for recurrence‐free survival after bacillus Calmette–Guérin treatment (multivariate).

	Reference	Including FOXP3‐positive cell density	
		HR (95% CI)	*p*
Age (years)			
≥ 72	< 72	1.28 (0.59–2.78)	0.529
Sex			
Male	Female	3.79 (0.48–29.78)	0.206
T stage			
Ta/T1 with Tis	Tis	0.88 (0.41–1.88)	0.740
Diagnosed condition			
New onset	Secondary onset	1.25 (0.53–2.94)	0.617
Smoking			
Yes	No	2.38 (0.96–5.90)	0.061
BCG maintenance therapy			
Yes	No	0.87 (0.24–3.17)	0.837
Prior intravesical chemotherapy			
Yes	No	1.94 (0.622–6.02)	0.254
FOXP3‐positive cell density			
≥ 300	< 300	3.84 (1.67–8.83)	0.002^a^

Abbreviations: CI, confidence interval; FOXP3, forkhead box P3; HR, hazard ratio.

^a^ *p* < 0.05.

### 3.5 FOXP3+/CD4+ Cell Ratio May Be a Better Biomarker for Predicting the Efficacy of BCG Treatment

Similarly, we evaluated whether the FOXP3+/CD4+ cell ratio could serve as a biomarker for predicting the efficacy of BCG treatment. Using a cut‐off value of 0.33, we divided 82 patients into high (*n* = 34) and low (*n* = 48) FOXP3+/CD4+ cell ratio groups. No significant differences were noted in patient characteristics between the two groups (Table 2). The mean RFS in the high FOXP3+/CD4+ cell ratio group was 50.8 months, shorter than that in the low FOXP3+/CD4+ cell ratio group (121.1 months), with a high statistical significance (*p* < 0.001) (Figure 6B). While recurrence patterns did not differ significantly, distant metastasis was observed only in the high FOXP3+/CD4+ cell ratio group (*p* = 0.454) (Table S2). Furthermore, in both univariate (HR: 3.78, 95% CI: 1.81–7.90, *p* < 0.001) (Table 3) and multivariate Cox regression analyses (HR: 3.82, 95% CI: 1.82–8.05, *p* < 0.001) (Table 5), a high FOXP3+/CD4+ cell ratio (≥ 0.33) was associated with a shorter RFS after BCG treatment. These findings indicate that the FOXP3+/CD4+ cell ratio prior to BCG treatment may be a better biomarker for predicting the efficacy of BCG treatment.

**TABLE 5:** Cox proportional hazard analysis for recurrence‐free survival after bacillus Calmette–Guérin treatment (multivariate).

	Reference	Including FOXP3+/CD4+ cell ratio	
		HR (95% CI)	*p*
Age (years)			
≥ 72	< 72	1.33 (0.58–3.03)	0.505
Sex			
Male	Female	3.02 (0.38–23.93)	0.294
T stage			
Ta/T1 with Tis	Tis	0.87 (0.40–1.87)	0.712
Diagnosed condition			
New onset	Secondary onset	1.26 (0.52–3.07)	0.610
Smoking			
Yes	No	1.71 (0.67–4.39)	0.263
BCG maintenance therapy			
Yes	No	0.52 (0.15–1.82)	0.308
Prior intravesical chemotherapy			
Yes	No	1.15 (0.38–3.45)	0.810
FOXP3+/CD4+ cell ratio			
≥ 300	< 300	3.77 (1.77–8.00)	< 0.001^a^

Abbreviations: CI, confidence interval; FOXP3, forkhead box P3; HR, hazard ratio.

^a^ *p* < 0.05.

## 4 Discussion

Here, we demonstrated that patients with bladder CIS who had relapsed after BCG treatment had a significantly higher peritumoral FOXP3‐positive cell density and/or FOXP3+/CD4+ cell ratio when assessed using pretreatment TURBT samples obtained from them. Moreover, patients with a FOXP3‐positive cell density > 300/mm^2^ or FOXP3+/CD4+ cell ratio > 0.33 exhibited significantly shorter intervals to relapse. These findings suggest that the FOXP3‐positive cell density and FOXP3+/CD4+ cell ratio may be biomarkers for predicting BCG treatment efficacy. Furthermore, while recurrence patterns exhibited no significant differences, distant metastasis was observed only in the high FOXP3+/CD4+ cell ratio group, suggesting a possible trend toward higher metastatic risk in the high group.

The distance between immune and tumor cells in the tumor immune microenvironment influences prognosis and therapeutic efficacy [10, 17]. In non‐small cell lung cancer, Tregs within 20 μm of the tumor cells were associated with a worse overall survival [18]. Additionally, a high density of programmed death‐ligand 1‐positive and CD8‐positive cells within 20 μm of the tumor cells was associated with better therapeutic outcomes in anti‐PD‐1 antibody therapy in melanoma [12]. Hence, we adopted a 20‐μm distance from tumor cells when counting the number of immune cells present.

In our study, the number of CD8‐positive T cells around the tumor cells did not correlate with the therapeutic effect of BCG treatment for bladder CIS (data not shown). Previous reports on NMIBC also indicated no association between the number of peritumoral CD8‐positive T cells and the therapeutic efficacy of BCG [19, 20]. Although the mechanism of action of BCG is not fully understood, a plausible pathway involved is the uptake of BCG antigen by antigen‐presenting cells and antigen presentation to CD4‐positive T cells. This process facilitates the activation of CD4‐positive T cells, followed by the release of Th1‐type cytokines from activated CD4‐positive T cells, ultimately contributing to an antitumor effect [21, 22]. In addition, a low Th1/Th2 ratio prior to BCG treatment has been reported to be associated with better BCG treatment efficacy [23]. Thus, both current and previous findings suggest that the status of CD4‐positive T cells, rather than the number of CD8‐positive T cells, around the tumor cells may influence the therapeutic effect of BCG.

Here, we demonstrated that a high density of FOXP3‐positive cells or a high FOXP3+/CD4+ cell ratio around tumor cells is associated with a poor response to BCG treatment for bladder CIS. Additionally, double immunostaining suggested that peritumoral FOXP3‐positive cells predominantly consist of CD4 + FOXP3+ positive cells. These cells act as Tregs with immunosuppressive properties, potentially inhibiting antitumor immunity [6]. Through this immunosuppressive effect, CD4 + FOXP3+ positive cells may contribute to BCG resistance. Indeed, a higher number of peritumoral Tregs has been reported to exacerbate the therapeutic effect of BCG for NMIBC [7]. Furthermore, a higher peritumoral FOXP3+/CD3+ cell ratio was associated with a worse prognosis in invasive bladder cancer [9]. Future research is needed to determine whether the FOXP3‐positive cell density or FOXP3+/CD4+ cell ratio could serve as the better biomarker.

Prior intravesical chemotherapy may influence FOXP3‐positive cell density. Among 13 patients who received intravesical chemotherapy before CIS diagnosis, no significant association was found with FOXP3‐positive cell density or the FOXP3+/CD4+ ratio. While previous studies reported chemotherapy‐induced reductions in circulating and peritumoral Tregs [24, 25], no such effect was observed here. Possible reasons include small sample size, differences between intravesical and systemic chemotherapy, or variability in the interval between chemotherapy and recurrence. Further studies are needed to clarify whether prior intravesical chemotherapy affects the predictive value of FOXP3‐positive cell density and the FOXP3+/CD4+ ratio for BCG treatment efficacy.

To the best of our knowledge, this is the first report evaluating the association between the immune cells present within 20 μm from the basement membrane at the site of bladder CIS and the efficacy of BCG treatment. Although there are numerous studies on the relationship between BCG treatment and immune cells in NMIBC, few have focused exclusively on bladder CIS, as done in the present study. Additionally, previous reports determined the number of immune cells at multiple tumor locations and used the average value measured [7]. Therefore, the present study differs from previous reports in that it focused only on bladder CIS and clearly defined the region where immune cells were counted.

This study has few limitations. First, this is a retrospective cohort study, and not a large number of patients were involved. Second, the number of patients who had received BCG maintenance treatment, which is now the standard, was small. Future studies with uniform treatment and follow‐up protocols may provide more robust results.

In conclusion, high FOXP3‐positive cell density or FOXP3+/CD4+ cell ratio around the tumor correlated with a worse response to BCG treatment for bladder CIS. Therefore, these two parameters, which can be measured from pre‐BCG treatment TURBT specimens, have potential as biomarkers for predicting the therapeutic efficacy of BCG in bladder CIS. Patients with higher values of these two parameters are likely at a higher risk of recurrence, including distant metastasis, and require more rigorous follow‐up.

## Author Contributions

**Yusuke Fukiage:** conceptualization, data curation, formal analysis, investigation, writing – original draft. **Akifumi Muramoto:** formal analysis. **Naoki Terada:** supervision. **Motohiro Kobayashi:** supervision, writing – review and editing.

## Ethics Statement

This study was approved by the Ethics Committee of the Faculty of Medical Sciences, University of Fukui (reference number 20200068; approved on July 21, 2020).

## Consent

Instead of obtaining individual written informed consent from patients, an opportunity to opt out was provided.

## Conflicts of Interest

The authors declare no financial support received for the present study. Naoki Terada is an Editorial Board member of the International Journal of Urology and a co‐author of this article. To minimize bias, he was excluded from all editorial decision‐making related to the acceptance of this article for publication.

## Supporting information


**Table S1.** Comparison of immune cells based on prior intravesical chemotherapy before bladder carcinoma in situ (CIS) diagnosis. This table presents the density of CD4‐, CD8‐, and FOXP3‐positive cells, as well as the FOXP3+/CD4+ cell ratio, in patients with and without prior intravesical chemotherapy. Statistical comparisons were performed using Student’s *t*‐test.

**Table S2.** Recurrence pattern after BCG treatment, separated by FOXP3‐positive cell density and FOXP3+/CD4+ cell ratio cut‐off values. Fisher’s exact test was applied to a 2 × 4 contingency table, and a single p‐value was calculated to assess overall differences across recurrence categories.
