# Supplementary material for: Peritumoral Infiltration of Regulatory T Cells Reduces the Therapeutic Efficacy of Bacillus Calmette–Guérin Therapy for Bladder Carcinoma In Situ
Source: Int J Urol. 2025 Mar 14;32(6):737–46. doi: 10.1111/iju.70044 (PMC12146248; doi:10.1111/iju.70044)
Supplement: Supplementary file 1 — Table S1. Comparison of immune cells based on prior intravesical chemotherapy before bladder carcinoma in situ (CIS) diagnosis. This table presents the density of CD4‐, CD8‐, and FOXP3‐positive cells, as well as the FOXP3+/CD4+ cell ratio, in patients with and without prior intravesical chemotherapy. Statistical comparisons were performed using Student’s t‐test. [file IJU-32-737-s002.docx]

**Table S1.** Comparison of immune cells based on prior intravesical chemotherapy before bladder CIS diagnosis

|  | Prior intravesical chemotherapy | |  |
| --- | --- | --- | --- |
|  | Yes (*n* = 13) | No (*n* = 69) | *p*-value |
| CD4-positive cell density (/mm^2^) | 1208 ± 877 | 1493 ± 1108 | 0.384 |
| CD8-positive cell density (/mm^2^) | 739 ± 527 | 702 ± 518 | 0.814 |
| FOXP3-positive cell density (/mm^2^) | 561 ± 683 | 384 ± 375 | 0.379 |
| FOXP3+/CD4+ cell ratio | 0.40 ± 0.23 | 0.29 ± 0.23 | 0.123 |

Statistical test: Student’s *t*-test

Abbreviations: FOXP3, forkhead box P3
