# Supplementary material for: Peritumoral Infiltration of Regulatory T Cells Reduces the Therapeutic Efficacy of Bacillus Calmette–Guérin Therapy for Bladder Carcinoma In Situ
Source: Int J Urol. 2025 Mar 14;32(6):737–46. doi: 10.1111/iju.70044 (PMC12146248; doi:10.1111/iju.70044)
Supplement: Supplementary file 2 — Table S2. Recurrence pattern after BCG treatment, separated by FOXP3‐positive cell density and FOXP3+/CD4+ cell ratio cut‐off values. Fisher’s exact test was applied to a 2 × 4 contingency table, and a single p‐value was calculated to assess overall differences across recurrence categories. [file IJU-32-737-s001.docx]

**Table S2.** Recurrence pattern (separated by cut-off values)

|  | FOXP3-positive cell density | | |  | FOXP3+/CD4+ cell ratio | | |
| --- | --- | --- | --- | --- | --- | --- | --- |
|  | Low | High | *p*-value |  | Low | High | *p*-value |
|  | (*n* = 10) | (*n* = 22) |  |  | (*n* = 11) | (*n* = 21) |  |
| Tis | 6 | 12 | 1.000 |  | 8 | 10 | 0.454 |
| Ta/T1 | 2 | 5 |  |  | 2 | 5 |  |
| Muscle invasive | 1 | 2 |  |  | 1 | 2 |  |
| Distant metastasis | 1 | 3 |  |  | 0 | 4 |  |

Statistical test: Fisher’s exact test

Abbreviations: FOXP3, forkhead box P3
